# Prostate-specific membrane antigen targeted gold nanoparticles for prostate cancer radiotherapy: does size matter for targeted particles?†
†Electronic supplementary information (ESI) available. See DOI: 10.1039/c9sc02290b


**DOI:** 10.1039/c9sc02290b

**Published:** 2019-07-18

**Authors:** Dong Luo, Xinning Wang, Sophia Zeng, Gopalakrishnan Ramamurthy, Clemens Burda, James P. Basilion

**Affiliations:** a Department of Radiology , Case Western Reserve University , Cleveland , OH , USA . Email: jxb206@case.edu; b Department of Biomedical Engineering , Case Western Reserve University , Cleveland , OH , USA; c Department of Chemistry , Case Western Reserve University , Cleveland , OH , USA . Email: burda@case.edu

## Abstract

Since the introduction of PSA testing, significantly more men have been diagnosed and treated for prostate cancer. Localized prostate cancer typically is treated with prostatectomy, however there is still a high risk of recurrence after surgery, and adjuvant radiation has been shown to mitigate disease progression. X-ray therapy is frequently used as an adjuvant to treat prostate cancer, but is an imperfect tool. In this report we describe the development of a targeted-radiosensitizing nanoparticle that significantly improves X-ray therapy. Taking advantage of the demonstrated radiosensitizing activity of gold nanoparticles (AuNPs) we developed targeted AuNPs and varied both surface ligand density and AuNP size to develop an optimized AuNP for X-ray radiotherapy. We conjugated a prostate-specific membrane antigen (PSMA) targeting ligand, PSMA-1, to AuNPs and found that the targeting ligand dramatically improved gold uptake by PSMA-expressing PC3pip cells compared with PC3flu cells lacking the PSMA receptors. Further, enhancement of radiotherapy was significantly more pronounced by internalization of smaller PSMA targeted-AuNPs. Our studies provide a foundation for design of size-selected AuNPs for targeted radiotherapy and, for the first time, systematically investigate both the effect of ligand and AuNP size on the cell uptake, tumor targeting and radiotherapy efficacy.

## Abstract 2

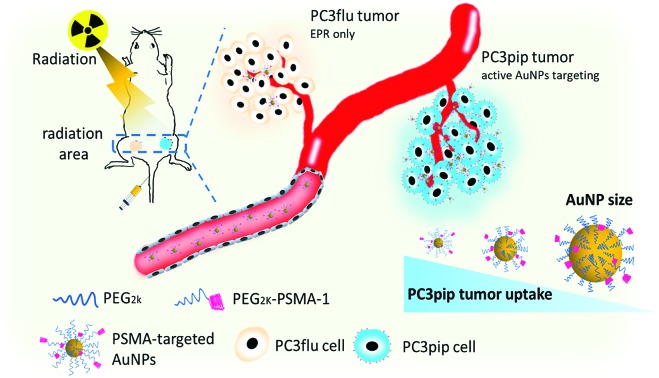
Prostate-Specific Membrane Antigen (PSMA) targeted radiosensitizers are developed for prostate cancer CT imaging and radiotherapy based on gold nanoparticles and a high-affinity targeting peptide, PSMA-1, revealing a size-dependent pattern.

## Introduction

Prostate cancer is the third most common non-skin cancer in the United States. In 2018, there were an estimated 164 690 new cases and 29 430 deaths from this disease.^1^ Localized prostate cancer typically is treated with surgery or radiation, and recurrent disease can be controlled temporarily with androgen ablation. However, almost all prostate carcinomas eventually become hormone refractory and then rapidly progress.^2^ Therefore, there exists a strong unmet clinical need for the development of therapies to more completely combat this deadly disease. Dependent on cancer stage, the correct treatment for each patient varies. For example, patients with slow growing, early prostate cancer may opt for watchful waiting. In contrast, an individual with early prostate cancer that is aggressively growing might choose surgery and radiation therapy.

Radiation therapy, also called X-ray therapy, uses high levels of radiation directed selectively to cancer tissues (whose location is usually defined by MRI) to kill prostate cancer cells. There, are however, side effects associated with this approach. These include erectile disfunction, urinary symptoms (*e.g.* bleeding and frequent urination) and symptoms deriving from irradiation of normal surrounding tissues, such as intestines, which can cause diarrhea. To more effectively utilize X-ray therapy, we and others have been developing radiosensitizing agents that can be targeted to diseased tissues and allow increased efficacy of radiotherapy with lower doses of X-rays.

Gold nanoparticles (AuNPs) show an outstanding versatility in biomedical applications, from drug delivery, imaging diagnostics, to photo- and radiation therapy.^3^–^8^ Upon X-ray irradiation, gold has a greater absorptivity over soft tissue and generates secondary electrons, producing biological damage, *e.g.* DNA strand break and mitochondrial dysfunction, to cells and tissue.^9^,^10^ This makes AuNPs outstanding for enhancement of radiation therapy. Advances in surface chemistry and nanotechnology have facilitated AuNPs development as radiosensitizers.^10^ AuNPs stabilized with PEG or zwitterions display excellent circulation *in vivo* and high accumulation in tumors *via* the enhanced permeability and retention (EPR) effect.^8^ Factors, such as surface coating and particle shape and size, also can influence radiotherapy outcomes.^10^ Spass *et al.* highlighted that increasing the PEG layer thickness on AuNPs could significantly impair radiosensitizing efficiency.^11^ Shape has a smaller effect on the radiosensitization achieved with AuNP.^12^,^13^ Among all the factors (coating, shape, composition, and size), size of the AuNPs may have the most profound impact on the success of radiotherapy.^14^

Generally, AuNPs interact with radiation through the photoelectric effect, in which radiation energy is absorbed and Auger-excited electrons are ejected from the gold. Photoelectric absorption depends on the atomic radius^15^,^16^ and gold with its large atomic size has an increased electron density and thus stronger absorption and X-ray radiation attenuation.^17^ Monte Carlo simulations^17^ showed, the bigger the particle size, the better the radiation enhancement. However, in reality the simulated size-dependent results do not always reflect the radiotherapy efficacy measured from *in vitro* and *in vivo* experiments. Studies by Dou *et al.* showed that the *in vitro* radiation enhancement is AuNP size- and concentration-dependent, with 13 nm particles showing the greatest ability to improve radiation therapy among a group of AuNP size ranging from 4–41 nm.^17^*In vivo* studies also indicated that 12 nm AuNPs led to better tumor inhibition upon radiation compared with 27.3 and 46.6 nm AuNPs.^18^ To date size investigations of AuNPs used untargeted nanoparticles that rely entirely on EPR for delivery. Until now, no study that systematically investigates how particle size and targeting affects radiosensitivity of prostate cancer *in vivo* has been reported.

Since AuNPs can significantly increase radiation damage in any tissue in which they are accumulated, it is essential to selectively target the AuNPs exclusively to the diseased tissues in order to be maximally exploited.^19^,^20^ Functionalizing AuNP sensitizers with targeting moieties can effectively avoid off-target accumulation and, thus, off-target tissue damage. In addition, if more AuNPs are efficiently delivered to the tumor, the overall radiation dose can be reduced, further reducing non-specific side effects of the therapy. Moreover, the photoelectric effect of AuNPs is very local and the Auger electrons released upon radiation have a limited penetration, further enhancing selective killing by reducing collateral damage to nearby un-targeted tissues.^21^ Antibodies and targeting ligands, such as folic acid and RGD, have been conjugated to AuNPs for targeted delivery with some success.^20^–^23^ However, some studies also showed that targeted AuNPs did not have significantly increased accumulation in tumor over untargeted particles, likely due to the low affinity of the targeting ligands,^24^ or an inability of antibody-targeted AuNPs to penetrate deeply into tumors, remaining only at peripheral tumor regions.^25^ Therefore, to identify a high affinity selective cancer biomarker and develop the matching highly-selective targeted gold nanoparticle is very attractive for radiotherapy.

Prostate-specific membrane antigen (PSMA) is a unique membrane-bound glycoprotein, which is overexpressed in prostate cancer and in the neovasculature of many solid tumors.^26^ Its expression increases progressively in higher grade prostate cancers, metastatic prostate cancer, and castration-resistant prostate cancer.^27^ PSMA is an excellent biomarker for prostate cancer and many PSMA targeted antibodies and ligands have been developed for imaging and treatment of prostate cancer.^27^ Recently, we have developed a urea-based PSMA targeting ligand (PSMA-1), which has a high binding affinity (2.01 nM) to PSMA.^28^ After conjugation to AuNPs, it can promote particle uptake and delivery of therapeutic molecules to prostate tumors, enhancing the therapy of prostate cancer.^29^

**Scheme 1:**
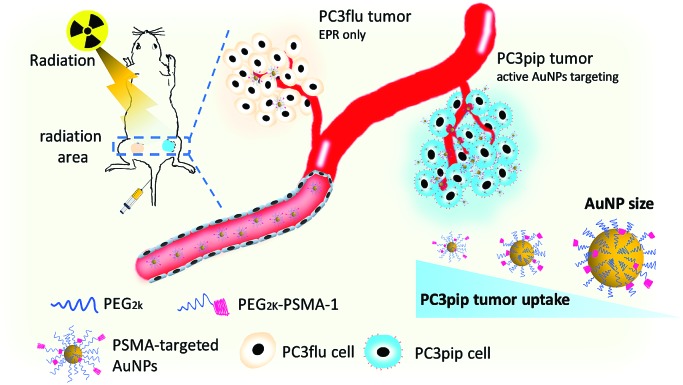
Schematic illustration of targeted radiotherapy of prostate cancer using PSMA-targeted AuNPs of different sizes.

In this work, we synthesized PSMA-targeted AuNPs with core sizes of 2 nm, 5 nm, and 19 nm and evaluated both the effect of the particle size and PSMA-1 ligand on the cell/tumor uptake and subsequent radiotherapy efficacy. We chose these three sizes to cover the range of NP sizes that have been previously tested to cause radiosensitivity. Conjugation of AuNPs with the PSMA-1 ligand dramatically improved AuNP uptake and led to radiation enhancement. *In vivo*, targeted AuNPs had a higher accumulation in PSMA-expressing PC3pip tumors than in PC3flu tumors, which do not express the PSMA receptor (Scheme 1). Our data suggests that the smallest PSMA-targeted AuNPs resulted in the greatest efficacy for prostate cancer radiotherapy at the same atomic concentration.

## Experimental section

### Synthesis and characterization of PSMA-targeted gold nanoparticles

PSMA-1 and SH-PEG_2k_-PSMA-1 were synthesized as reported previously with slight modification.^27^ Gold nanoparticles with three different sizes were synthesized using a reported protocol.^11^ The detailed procedure is described in the ESI.† The concentration of the AuNP samples was determined by UV-vis spectroscopy based on the plasmonic absorption band at 520 nm (TECAN, infinite M200). To functionalize AuNPs with SH-PEG_2k_-PSMA-1, a 1000 molar excess of SH-PEG_2k_-PSMA-1 and SH-PEG_2k_ ligand at different molar ratios (1 : 8, 1 : 4, 1 : 1 to 2 : 1) was added to react with 1 equivalent of AuNP-DDA or AuNP-citric acid for 2 days. Excess unreacted ligands were removed by extensive purification using centrifuge filters (MWCO = 30 kDa, GE Healthcare). As control, non-targeted AuNPs were also prepared in the same way with only SH-PEG_2K_ added and purified.

The hydrodynamic size of AuNPs was characterized with a dynamic light scattering system (DynaPro NanoStar). The zeta potential of AuNPs was measured with a Zetasizer Nano (Malvern). For absolute size determination, transmission electron microscopy (FEI Tecnai F300 kV) was used. To visualize the polymer shells, the nanoparticles loaded copper grids were stained by one drop of 2% phosphotungstic acid for 5 min and then the excess liquid was wicked off with filter paper. After staining, the grids were dried again in air before TEM testing. Gel electrophoresis for all the PSMA-targeted and untargeted nanoparticles with various sizes were performed on 1% agarose gel and 1× TAE running buffer at 120 kV. Each chamber was loaded with 10 μL of 2 μM AuNPs, 5 μL of glycerol, and 5 μL of 4× TAE.

### Quantification of AuNP uptake by cells

PC3pip (PSMA+) cells and PC3flu (PSMA–) cells were seeded in 6-well plates at 1 × 10^5^ cells per well and cultured for 24 h. Then AuNPs were added to each well at Au concentration of 60 μg ml^–1^ and co-incubated for 1, 6, 24 h. The medium was removed and the cells were washed three times with PBS. Next, the cells from each well were trypsinized, centrifuged, washed with PBS, counted and collected in 1.5 ml Eppendorf tubes. 0.5 ml aqua regia (HCl : HNO_3_ at 3 : 1) was added to each tube to digest the cells overnight. Each sample was then diluted with DI water. Au concentration in each sample was measured with ICP-MS (Agilent technologies, 700 series).

### Silver staining assay

PC3pip and PC3flu cells were seeded in 8-well plates at 2000 cells per well and AuNPs were added to each well at Au concentration of 60 μg ml^–1^, and co-incubated for 1, 6, 24 h. The cells were then stained with silver as reported previously.^29^ The cells were observed under a Leica DM4000B fluorescence microscope (Leica Microsystem Inc.).

### *In vitro* radiosensitization evaluation

PC3pip and PC3flu cells were seeded in 96-well plates at 1 × 10^4^ cells per well and incubated overnight. Both the PSMA-targeted and untargeted AuNPs of different sizes were added at an Au concentration of 60 μg ml^–1^. Cells without any particles were used as a control. Following an incubation for 24 h, the medium was removed and cells were washed with PBS to remove the non-internalized AuNPs. The cells were then irradiated with X-ray at doses of 0, 2, 4, 6 Gy and incubated for another 24 h. All treatments were carried out only once. Cell viability was determined with a CCK8 assay by adding 10 μl CCK8 agents to each well and measuring the absorbance at 450 nm after 4 h incubation (TECAN, infinite M200).

For the colony formation assay, PC3pip and PC3flu cells with all the same treatments were trypsinized, counted, and seeded in the 6-well plates. Following an addition incubation of 10 days, the colonies formed were washed with PBS and fixed with 4% paraformaldehyde. A 0.4% crystal violet solution in PBS was added for colony staining. The colony number was then counted to calculate the surviving fraction.

### Animals and tumor xenograft models

All animal studies were performed in compliance to relevant laws and guidelines and were approved by Case Western Reserve University's IACUC (Animal Experimentation application 2015-003, approved 3/27/2018–3/27/2021). Nude mice with flank tumors, PC3pip tumor at the right side and PC3flu tumor at the left side, were used to evaluate the radiotherapy effect of PSMA-targeted AuNPs. PC3pip or PC3flu cells were prepared and suspended in PBS/matrigel at 1 × 10^7^ cells per ml. The nude mice were anesthetized under isoflurane and inoculated with 100 μL cell suspension subcutaneously. Animals were observed every day until tumors achieved a size of approximately 100 mm^3^.

### *In vivo* CT imaging

Mice bearing PC3pip and PC3flu tumors were randomly picked and intravenously injected with PSMA-targeted and un-targeted AuNPs with core size of 2 nm, 5 nm and 19 nm at a dose of 5 mg Au per kg. The mice were anesthetized under isoflurane and scanned by a preclinical Siemens Inveon positron emission tomography-computed tomography system before and 0.5 h, 1 h, 2 h, 4 h, 6 h and 24 h after intravenous injection of AuNPs. The CT scanning was performed at a tube voltage of 70 kV, current of 300 μA, and gantry rotation time of 140 ms. CT images were reconstructed and the Hounsfield Unit (HU) was quantified at the tumor areas.

### Biodistribution

At 24 h postinjection of the AuNPs, the mice were sacrificed after the last CT scanning. PC3pip and PC3flu tumors and organs, including liver, spleen, heart, lung, kidneys, and urine, were discretized, weighed and lyophilized. The dried samples were then immersed in aqua regia and digested with gentle shaking for 3 days. When all the tissues were completely digested, the aqua regia solution was diluted with DI water and then measured with ICP-MS to determine the Au content.

### *In vivo* radiation therapy

When the tumor size reached about 100 mm^3^, the tumor-bearing mice were divided randomly into groups, which were injected with PBS, PSMA-targeted AuNPs with core size of 2 nm, 5 nm and 19 nm, un-targeted AuNPs with core size of 2 nm, 5 nm and 19 nm, respectively. The Au dose for each group was 5 mg kg^–1^. 4 hours after injection, the mice received 6 Gy of X-ray radiation focused onto the tumor area. X-ray radiation was given only once, then all irradiated mice were monitored for tumor sizes and body weight every other day over 20 days. A control group of mice were injected with PBS, PSMA-targeted AuNPs and un-targeted AuNPs with core size of 2 nm, 5 nm and 19 nm, but they received no X-ray irradiation. Tumor growth was also monitored at the same time points.

### Statistics

All the experiments were performed in triplicates unless stated otherwise. All numerical results are expressed as mean ± SD. Descriptive statistics and significant differences between groups were analyzed using two-tailed Student's *t*-tests, and the difference was considered significant if *p < 0.05 and **p < 0.01.

## Results

**Fig. 1:**
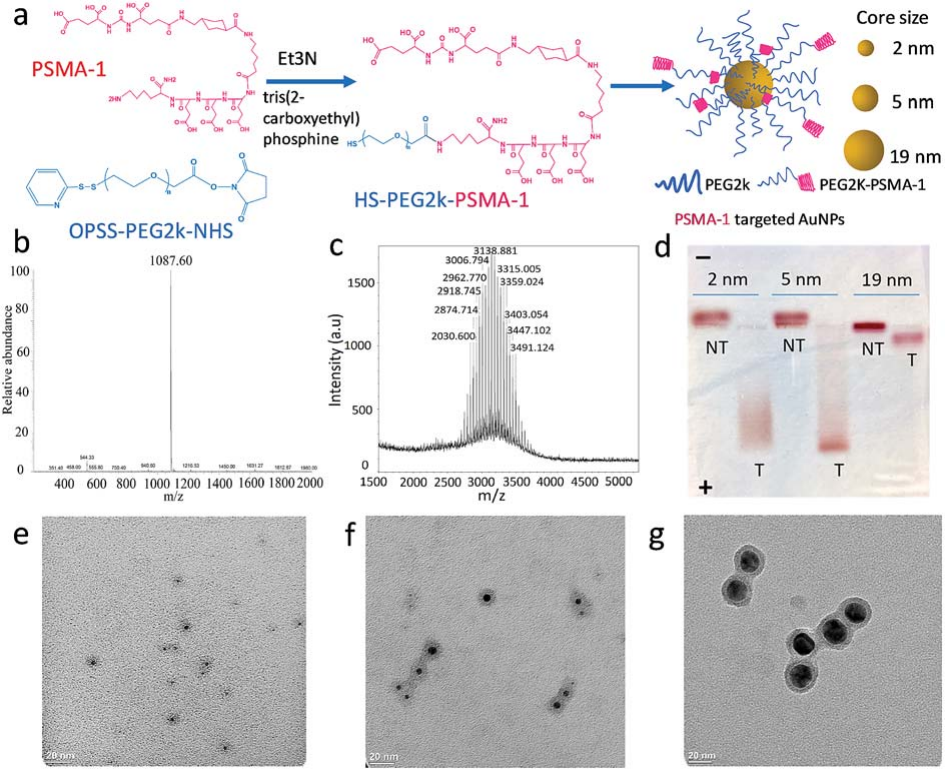
Synthesis of AuNPs with PSMA-1 targeting. (a) Molecular structure of PSMA-1 conjugated PEG_2k_ (PSMA-1 in pink and PEG_2k_ in blue), and schematic of AuNPs modified with both PEG_2k_-PSMA-1 and PEG_2k_; (b) ESI-MS spectrum of PSMA-1 with *m*/*z* at 1087; (c) MOLDI-TOF mass spectrum of PEG_2k_-PSMA-1 conjugations; (d) Agarose gel electrophoresis demonstrates the successful binding of PEG_2k_-PSMA-1 to AuNPs and mobility of AuNPs with different sizes (T, PSMA-targeted AuNPs; NT, nontargeted AuNPs); (e–g) TEM images AuNPs with average core sizes of (a) 2 nm, (b) 5 nm and (c) 19 nm, with PEG_2k_ shell stained with phosphotungstic acid.

The first step in these studies was to synthesize three different sizes of AuNPs (core sizes of 2 nm, 5 nm and 19 nm) and derivatize them with PSMA-1 (Fig. 1a and S1†). We first synthesized dodecylamine stabilized 2 nm and 5 nm AuNPs and citric stabilized 19 nm AuNPs. To provide better bioavailability each set of AuNPs was then PEGylated with PEG_2k_*via* ligand-exchange.^30^ To facilitate active targeting PSMA-1-PEG_2k_-SH was also included in the PEGylation. PSMA-1 with a calculated molecular weight of 1087 was synthesized using a solid phase synthesis and characterized by its ESI-MS spectrum (Fig. 1b). We then conjugated OPSS-PEG_2k_-NHS to PSMA-1 ligands *via* an amide linkage (Fig. 1a and S1†) and later removed the OPSS, producing PSMA-1-PEG_2k_-SH. The successful conjugation was confirmed by MALDI-TOF MS spectrometry: approximately 1 kDa mass shift was observed (Fig. 1c).

Next, we conjugated PSMA-1-PEG_2k_-SH and mPEG_2k_-SH (at a ratio of 1 : 4) to AuNPs to enable active targeting. For the untargeted control, the same amount of mPEG_2k_-SH was reacted in the absence of PSMA-1-PEG_2k_-SH. Due to the abundant glutamic acid residues of PSMA-1 ligands, the PSMA-targeted AuNPs are negatively charged, while the untargeted AuNPs were neutral. We performed agarose gel electrophoresis to demonstrate the successful functionalization of AuNPs with PSMA-1 ligand. As displayed in Fig. 1d, all the PSMA-targeted AuNPs migrated to the anode whereas the untargeted AuNPs showed marginal migration to the cathode, indicating that AuNPs were successfully functionalized with PSMA-1. The 2 nm and 5 nm PSMA-targeted AuNPs moved much farther within 30 min electrophoresis than the 19 nm particles. This is due to the size dependent frictional drag forces within the agarose gel.^31^ Based on DLS measurements, the hydrodynamic diameters for the AuNPs with core size of 2 nm, 5 nm and 19 nm were 10.6 nm (PDI = 0.14), 19.4 nm (PDI = 0.28) and 26.4 nm (PDI = 0.10), respectively. The *ζ*-potential of the AuNPs is another possible explanation for differences in the migration distances.^32^ The PSMA-targeted 2 nm and 5 nm AuNPs showed a similar negative charge of –32.17(±0.31) mV and –32.92(±1.22) mV. In contrast, 19 nm AuNPs had a *ζ*-potential of –24.83(±0.45) mV. Since the PSMA-1-PEG_2k_-SH was conjugated to differently sized AuNPs at the same ratio, the difference of apparent *ζ*-potential was mainly due to the increase of the hydrodynamic size and screening by the AuNP core.^33^ The actual total PEG_2k_ coverage for 5 nm AuNPs is 800 per particle, with ligand density of 7.7 PEG nm^–2^.^34^ For 2 nm and 19 nm AuNPs, according to their surface area compared to 5 nm AuNPs, the number of ligands on each particle surface would be about 96 and 8728. By measuring the remaining PSMA-1-PEG_2k_ from particle conjugation, the PSMA-1 number per particle for 2 nm, 5 nm and 19 nm AuNPs is about 26, 140 and 2022, respectively (Fig. S2†).

Final characterization of the AuNPs was performed using TEM to measure their absolute core sizes. As demonstrated in Fig. 1e–g, all the AuNPs samples showed unimodal distribution without any aggregation. The core sizes for the three AuNPs samples were 2.2(±0.6) nm, 4.8(±1.4) nm and 19.0(±2.1) nm. The tight symmetrical size distribution of the AuNP samples is also reflected in their narrow plasmonic resonance spectra, (Fig. S3†). To determine the PEGylation shell thickness using TEM we stained the PEGylated AuNPs with 2% phosphotungstic acid (PTA) before electron microscopy. Compared to the AuNP core, the PTA stained shells were lighter in color, allowing observation of the PEG corona. By measuring the diameter of the PEG corona of each sample, the actual sizes of the three samples were 8.7(±1.3) nm, 13.9(±2.8) nm and 25.7(±2.2) nm, respectively. The PEGylation of the AuNPs led to a consistent increase in particle diameter of about 7 nm. The distribution of the core and overall sizes is plotted in Fig. S4.† It is interesting to notice that the hydrodynamic sizes measured by DLS were slightly larger than that measured using the stained TEM images, which is due to the interaction between the particles and the aqueous environment. Recently, we have demonstrated that PEGylation of AuNPs provides stability for more than one year.^29^

**Fig. 2:**
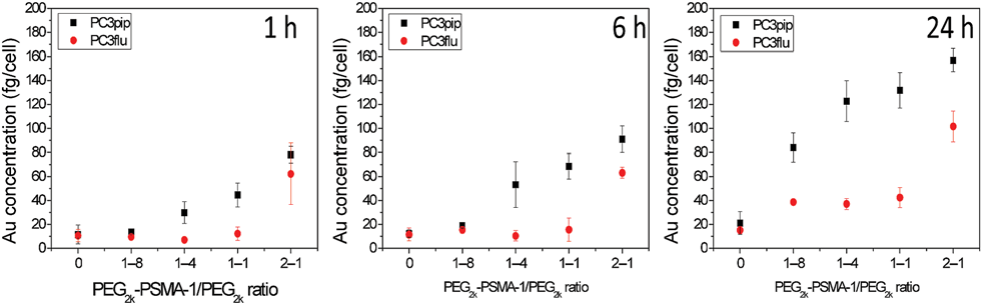
PSMA-1 ligand mediated cell uptake of 5 nm AuNPs. AuNPs were conjugated with PEG_2k_-PSMA-1 and PEG_2k_ at ratios of 0, 1 : 8, 1 : 4, 1 : 1 and 2 : 1. The Au content in each cell was verified as a function of PSMA-1 conjugation ratio as determined by ICP-MS. Data are presented as mean ± SD (*n* = 3).

Though the PSMA-1 has a superior binding affinity over the most commonly used RGD ligands,^35^,^36^ an excess conjugation of targeting moiety on the AuNP surface is not always helpful.^5^ To evaluate the ability of the PSMA-1 ligand to promote active AuNP uptake and to optimize its conjugation level, we used 5 nm AuNPs, and grafted PSMA-1-PEG_2k_-SH and mPEG_2K_-SH at different ratios of 1 : 8, 1 : 4, 1 : 1 and 2 : 1. After formation and characterization of the particles as described above, we incubated the AuNPs with PSMA-expressing PC3pip cells or PSMA-negative PC3flu cells for different periods of time. These studies showed that increasing the amount of PSMA targeting moiety grafted on AuNPs surface resulted in an increase in selective uptake of AuNPs by PC3pip cells at both the 1 : 4 and 1 : 1 ratios of PSMA-1-PEG_2k_-SH/mPEG_2K_-SH with the greatest uptake measured at 24 h (Fig. 2). The highest conjugation ratio, 2 : 1, of PSMA-1-PEG_2k_-SH/mPEG_2K_-SH did not result in differential selective uptake between PC3pip and PC3flu cells. Therefore, we conjugated all AuNPs with size of 2 nm, 5 nm and 19 nm with 1 : 4 (20%) PSMA-1 targeting and used them in all studies.

**Fig. 3:**
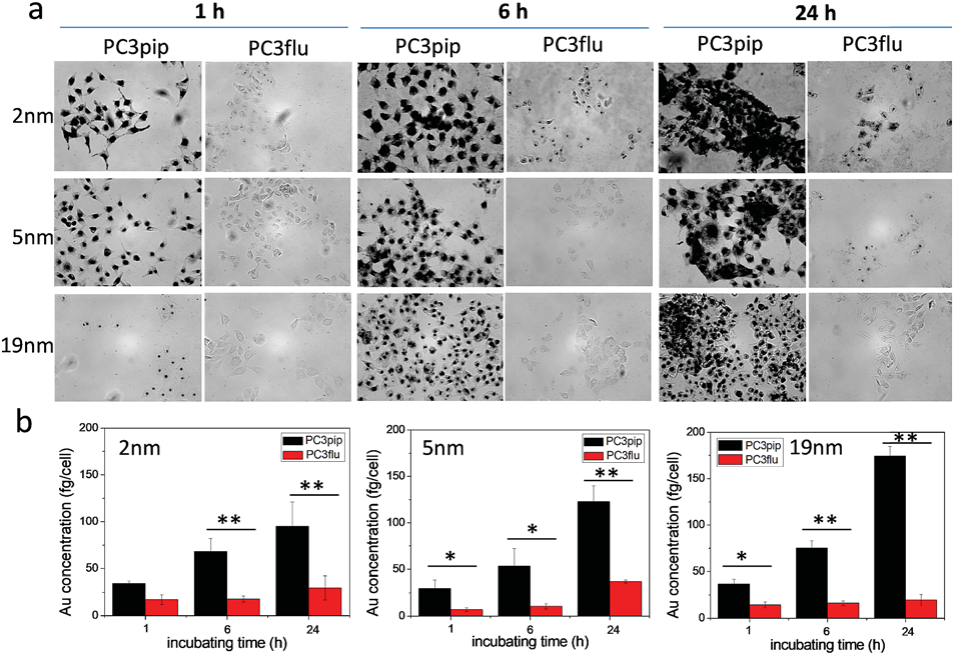
Effect of particle size on cell uptake of the PSMA-targeted AuNPs. (a) PSMA-targeted AuNPs with core sizes of 2 nm, 5 nm and 19 nm were incubated with PC3pip and PC3flu cells for 1 h, 6 h and 24 h and then stained by silver staining kits. The silver nucleates around AuNPs and thus reveals AuNP uptake by the cells. (b) Quantitative Au content in cells incubated with 2 nm, 5 nm and 19 nm AuNPs as determined by ICP-MS divided by total cell number. Data are presented as mean ± SD (*n* = 3), and differences between groups are compared with two-tailed *t*-tests, *p ≤ 0.05 **p ≤ 0.01.

We next investigated the effect of AuNP size on cellular uptake by incubating both PSMA-targeted and untargeted AuNPs with PC3pip and PC3flu cells for 1 h, 6 h, and 24 h and visualizing the AuNP by silver staining. As Fig. 3a shows, PSMA expressing PC3pip cells exhibited a temporal and selective uptake of the targeted AuNPs. We also observed that cells incubated with PSMA-targeted AuNPs having a core size of 2 nm exhibited much more silver staining than 5 nm and 19 nm particles at all time points. The silver staining was more homogeneous for each individual cell with 2 nm AuNPs than with 5 nm and 19 nm particles. For 19 nm AuNPs, the staining was mostly located in the center of cells at 1 h. As a control, untargeted AuNPs of the same sizes were also tested and showed no selective uptake by either PC3pip or PC3flu cells (Fig. S5†).

To quantify the AuNPs uptake by PC3pip and PC3flu cells, we performed ICP-MS measurements. Cells incubated were analyzed for gold content (Fig. 3b). The amount of atomic gold measured in PC3pip cells incubated with PSMA-targeted AuNPs increased with time and was significantly higher than that measured in PC3flu cells. For the untargeted AuNPs, both cell lines shared a similar low uptake (Fig. S6†). AuNP size also influenced PC3pip cell uptake of targeted nanoparticles. In contrast to silver staining, at 24 h there was a significant higher uptake of 19 nm PSMA-targeted AuNPs (174.3 ± 10.3 fg per cell) compared to either the 5 nm (122.8 ± 17.1 fg per cell) or 2 nm (95.1 ± 25.9 fg per cell) targeted-AuNPs (*p* ≤ 0.05). There was no significant difference between 2 nm and 5 nm AuNPs uptake. Interestingly, the silver staining indicated a much higher level of silver precipitating in PC3pip cells incubated with 2 nm AuNPs than cells with 5 nm and 19 nm AuNPs. This is likely due to the fact that at similar concentrations of Au, the smaller diameter particles are greater in number compared to larger AuNPs providing more sites for silver ions to nucleate and deposit on their surface, resulting in greater contrast. This depositing and autocatalytic reaction amplifies and produces a large amount of silver precipitation in cells^37^ that is not quantitative for the amount of gold atoms contained by the cells. Therefore, we observe much darker staining for the 2 nm and 5 nm AuNPs than for the 19 nm AuNPs, though the actual atomic gold content was lower for the 2 nm and 5 nm AuNPs than for the 19 nm AuNPs.

**Fig. 4:**
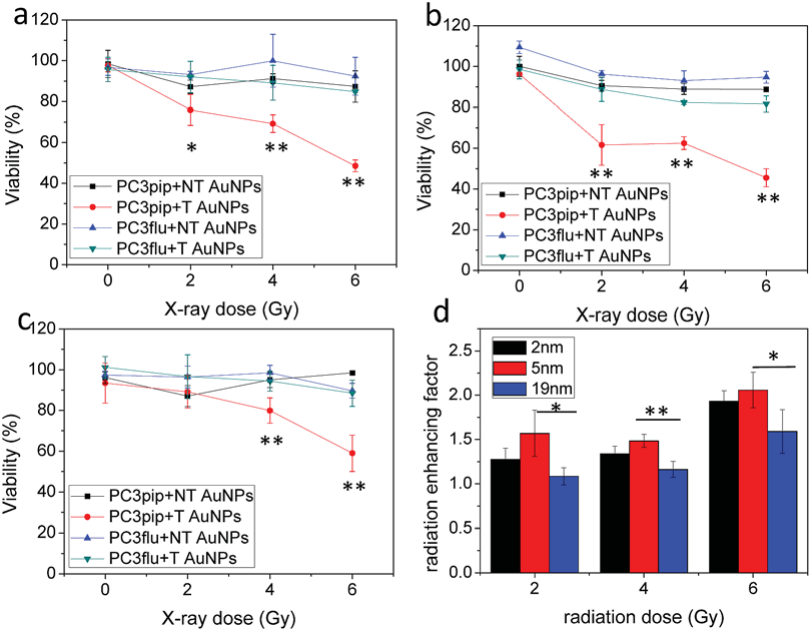
*In vitro* radiation enhancement by AuNPs with different sizes. PC3pip and PC3flu cells were incubated with both PSMA-targeted (T) and non-targeted (NT) AuNPs with sizes of (a) 2 nm, (b) 5 nm and (c) 19 nm for 24 h and irradiated with X-ray at 2 Gy, 4 Gy and 6 Gy. (d) Radiation enhancing factor (REF, ratio of eradicated cells with and without AuNPs) for PC3pip cells incubated with PSMA-targeted AuNPs at radiation doses of 2 Gy, 4 Gy and 6 Gy. Data are presented as mean ± SD (*n* = 3), and differences between groups are compared with two-tailed *t*-tests, *p ≤ 0.05 **p ≤ 0.01.

We next studied the combination of PSMA targeting and different particle sizes on radiation enhancement. We incubated PC3pip and PC3flu cells with a very low dose of PSMA-targeted and untargeted AuNPs for 24 h and then exposed them to either 2 Gy, 4 Gy or 6 Gy dose of X-rays. The particles alone, without any irradiation, did not cause any toxicity to the cells (Fig. 4a–c, 0 Gy). Increasing irradiation of the cells from 2 Gy to 6 Gy resulted in a dramatic decrease in cell viability for PC3pip cells treated with PSMA-targeted AuNPs of all sizes, while the PC3flu cells showed marginal reductions in viability. Without PSMA targeting, the AuNPs also had limited radiation enhancement for either PC3pip or PC3flu cells, the viability of which was similar to cells without AuNPs irradiated at the same doses (Fig. S7†). It became evident that PSMA targeting could enhance the radiation effect by promoting cell specific uptake of AuNPs. To compare the effect of particle size on radiosensitization, the radiation enhancing factor (REF), defined as ratio of eradicating cells with and without AuNPs, was calculated at all radiation doses for all particle sizes.^23^Fig. 4d indicates a better radiation enhancement for smaller particles, despite an opposite trend in cellular gold content. In contrast, among all the non-targeted AuNPs of different sizes, the REF approximately equaled 1, indicating little to no enhancement (Fig. S8†). A colony formation assay was also conducted for PC3pip and PC3flu cells after treatment. Above 2 Gy radiation all AuNPs negatively impacted colony formation, however, PSMA-targeted AuNPs showed a decrease in colony formation compared to all other controls at all radiation doses (Fig. S9†). The fact that non-targeted AuNPs did not affect viability of cells *in vitro* suggests that colony formation may be more sensitive to irradiation than the cellular viability assay. The sensitization enhancement ratio (SER) for PC3pip cells treated with 2 nm, 5 nm and 19 nm PSMA-targeted AuNPs was determined to be 2.4, 2.1, and 1.7, respectively, according to the survival fraction curves generated from the colony formation assay (Fig. S9†).^38^

**Fig. 5:**
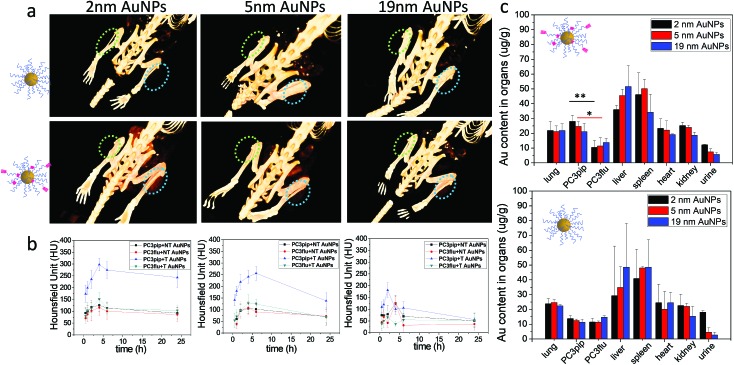
Tumor uptake and biodistribution of AuNPs with different sizes. (a) *In vivo* 3D CT images of PC3pip (blue circle) and Pc3flu (green circle) tumor-bearing mice at 4 h post-injection of both PSMA-targeted and untargeted AuNPs. (b) Quantitative analysis of the CT signals at PC3pip and PC3flu tumor regions after injection of PSMA-targeted (T) and non-targeted (NT) AuNPs at 0.5 h, 1 h, 2 h, 4 h, 6 h and 24 h, (c) biodistributions of AuNPs after 24 h as determined by ICP-MS. Data are presented as mean ± SD (*n* = 3), and differences of Au content in PC3pip and PC3flu tumors are compared with two tailed *t*-tests, *p ≤ 0.05 **p ≤ 0.01.

To investigate active tumor targeting of AuNPs compared to passive tumor accumulation and the influence of particle size on particle accumulation, we subcutaneously injected both PC3pip and PC3flu cells at the right and left flanks of nude mice. When the tumor reached approximately 100 mm^3^, either PSMA-targeted or untargeted AuNPs of various sizes were intravenously injected *via* the tail vein, and CT scanning was conducted before and at 0.5 h, 1 h, 2 h, 4 h, 6 h, and 24 h post particle injection. Volume-rendered 3D images were reconstructed and are shown in Fig. 5a. Generally, it is known that nanoparticles accumulate in tumors due to the EPR effect.^5^ In our system, however, CT measurements demonstrated that accumulation was enhanced for PSMA-targeted AuNPs. Comparing the PC3pip tumor with the PC3flu tumor in the same mouse (4 h), a pronounced CT signal enhancement was observed, indicating more PSMA-targeted AuNP deposition in PSMA-expressing PC3pip tumors than in PSMA-negative PC3flu tumors. In contrast, for mice injected with untargeted AuNPs, there was no measurable CT signal enhancement in the PC3pip tumors over the PC3flu tumors, suggesting no active targeting to the tumors. Comparing the influence of size on tumor uptake of the nanoparticles indicated that the PSMA-targeted 2 and 5 nm AuNP accumulated significantly more nanoparticle in the PSMA-positive tumors than did the PSMA-targeted 19 nm particles (Fig. 5a and b). Though, bigger AuNPs have higher X-ray attenuation (Fig. S10†), the *in vivo* tumor CT imaging is predictive of AuNP concentration within the tumors (Fig. 5b and c).

Twenty-four hours after injection, we sacrificed the mice and analyzed the Au content in the PC3pip and PC3flu tumors and the main organs using ICP-MS (Fig. 5c). For mice injected with PSMA-targeted AuNPs, there was a significantly higher Au content in PC3pip tumors than in the PC3flu tumors. The PC3pip/PC3flu ratio 2.8, 2.2 and 1.5 times for particles with 2 nm, 5 nm and 19 nm diameter, respectively. In mice injected with untargeted AuNPs the Au concentration was the same in both types of tumors and similar to the amount in PC3flu tumors of mice injected with targeted particles. Despite the active targeting of AuNPs to PC3pip tumors, the max tumor uptake was still in the liver and spleen. All the AuNP systems used in our study had a hydrodynamic diameter bigger than the renal clearable threshold of 6 nm, thus they would be taken up in the reticuloendothelial system (RES), mostly liver and spleen.^39^ However, the smallest AuNP (2 nm) did show partial excretion *via* the urine with the average Au content lower in the liver and higher in the urine than either the 5 or 19 nm particles. The CT images also displayed temporarily obvious Au accumulation in the bladder for mice injected with 2 nm AuNPs (Fig. S11†).

**Fig. 6:**
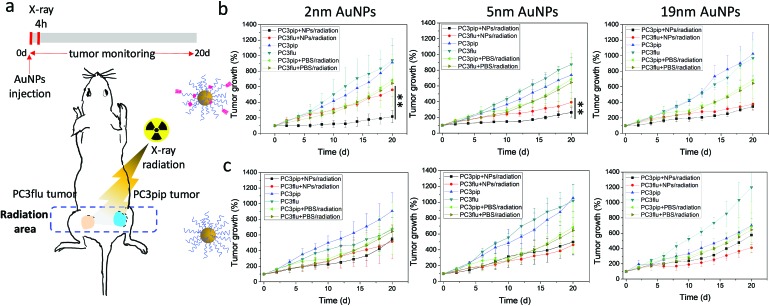
*In vivo* AuNP enhanced radiotherapy. (a) Schematic PC3pip and PC3flu tumor bearing mouse, and X-ray radiation of tumors and timeline. (b and c) PC3pip and PC3flu tumor growth curves after injection of (b) PSMA-targeted and (c) untargeted AuNPs with different sizes. Data are presented as mean ± SD (*n* = 3), and growth inhibition of PC3pip and PC3flu tumors are compared with two-tailed *t*-test, **p ≤ 0.01.

To explore the potential of the PSMA-targeted AuNPs to enhance prostate cancer radiotherapy, mice bearing both PC3pip and PC3flu tumors were injected with either targeted or untargeted AuNPs and X-ray irradiated at the peak particle accumulation (4 hours) with 6 Gy. Tumor growth and body weight were monitored for 20 days and compared with mice receiving the same injection but no irradiation. To minimize the X-ray effect on the rest of the body, we restricted the radiation exposure to only the tumor (Fig. 6a).

In all cases, irradiation of the tumor-containing flank regions resulted in a reduction in tumor growth compared to animals that did not receive irradiation, and AuNPs could enhance the radiation comparing to mice injected with only PBS (Fig. 6b and c). Comparing the growth curves of PC3pip and PC3flu tumors from animals that received targeted AuNPs with and without irradiation, only the targeted 2 and 5 nm AuNPs showed enhanced X-ray therapy in tumors that expressed the PSMA biomarker, *i.e.* PC3pip, with the greatest attenuation resulting from the 2 nm AuNPs. Interestingly, the 19 nm targeted AuNPs did not show a difference in growth rate between PC3pip and PC3flu cells, which were both lowered. Tumor sizes in mice receiving different particles and radiation at Day 20 was showed in ESI Fig. S12.† In animals that received non-targeted AuNPs, there was less radiosensitization and no difference in growth attenuation among any of the different sized AuNPs. Without radiation, both the PC3pip and PC3flu tumors from all the AuNPs treatments shared similar growth kinetics, which was the same as when mice were administered PBS (Fig. S13†). Body weight of all mice under different treatments did not show significant changes (Fig. S14†), indicating that it is safe to use AuNPs as radiosensitizer for prostate cancer therapy.

## Discussion

Active targeting of AuNPs with ligands to enable a high local accumulation in tumor tissues has been extensively investigated for non-invasive imaging and delivery of chemotherapeutics.^40^ Recently, there have been several studies demonstrating the potential utility of AuNPs to serve as radiosensitizers for tumors in different mouse models.^10^ In this study we utilize PSMA-targeted AuNPs and show significant active targeting of AuNPs within tumors expressing PSMA receptors. The gold uptake of the targeted particles is greater than that seen from passive accumulation alone, and significantly impacts the efficacy of X-ray therapy in mouse models of prostate cancer.

For radiotherapy enhancement using targeted delivery of AuNPs, which achieves selective localization to cancer tissues, is essential to avoid off-target damage to normal tissue. We constructed an AuNP to serve as a radiation therapy sensitizer by synthesizing a gold nanoparticle core and then PEGylating the AuNP surface. The PEG was included to reduce nonspecific binding and enhance *in vivo* circulation time.^30^ Different amounts of PSMA-1 ligand were conjugated to the outermost region of the PEG with the aim of targeting the particles to the PSMA biomarker. The results indicate that the conjugated PSMA-1 served as a strong ligand for PSMA receptor and significantly increased the uptake of the PSMA-1-labeled AuNPs. However, AuNP uptake did not increase linearly with increasing levels of PSMA-1 conjugated to the surface of the AuNP. One possibility for this finding is that an excessive amount of PSMA-1 ligands on the NP surface may prolong the interaction between PSMA-1 ligands and the receptors on cell surface, and delay the internalizing process.^41^ Further, AuNPs with high levels of PSMA-1 ligands may bind many receptors limiting access for other particles to bind to the cells. A very similar phenomenon was observed when conjugating a cathepsin B inhibitor to the AuNP surface, where a maximum activity was achieved when only 10% of the PEG on the AuNP was grafted with the inhibitor.^5^ At highest PSMA-1 conjugation ratio, 2 : 1, there are excess PSMA-1 on nanoparticle surface, which does not provide further binding efficacy. However, the additional ligands consume extra receptors decreasing the number of available ‘landing spots’ for forthcoming nanoparticles. The excessive receptor binding to a larger entity prevents receptor-mediated internalization, which does not occur when the ligand density on the particle is lower.^42^ In our studies, AuNPs with 20% PSMA-1 grafted to the PEG surface coating on the particles showed the greatest selective uptake of the targeted AuNPs by PC3pip cells compared to PCflu cells (that do not express PSMA receptor).

We and others have found that AuNP size has a profound influence on cellular uptake and *in vivo* bio-kinetics.^43^ Previous uptake studies using untargeted AuNPs showed that a maximum uptake by mammalian cells *in vitro* occurred with particles of 50 nm in diameter, while *in vivo* uptake was optimal for 12 nm particles.^44^ It is thought that AuNPs enter cells *via* the endocytosis pathway.^45^,^46^ In this present study, we compare for the first time how the combination of targeting ligands and particle size impacts cell uptake both *in vitro* and *in vivo*. Our results show that AuNPs with a core size of 2, 5, or 19 nm, did not appreciably affect uptake when the AuNPs did not possess a targeting ligand. However, at the optimal concentration of PSMA-1 of the AuNP surface, the levels of gold, measured by ICP, within the cells expressing the PSMA receptor increased with increasing core diameter for *in vitro* studies.

Targeted-AuNP uptake was also slightly more rapid for the larger 19 nm particles. When the PSMA-1 ligand binds to receptors on prostate cancer cells, the cell membranes will invaginate and finally engulf the whole particle. According to Chan *et al.*, this “wrap” process is determined by factors of adhesion and membrane stretching, and the bending energy of the membrane.^41^ Nanoparticles with a size of about 55 nm demonstrated the fastest wrapping time and they could be engulfed individually, which enabled the fastest uptake. Smaller nanoparticles must be clustered together before uptake, resulting in a delayed uptake.^41^ This theory may also explain the slightly faster uptake of targeted 19 nm AuNPs *in vitro*. Our *in vivo* results, however, revealed a reversed size-dependent pattern of tumor uptake with the highest tumor uptake measured for 2 nm PSMA-targeted AuNPs. This may be explained by the fact that small nanoparticles are more likely to extravasate through porous/leaky tumor neovasculature than larger nanoparticles and thus can penetrate deep into tumors.^47^,^48^ Furthermore, it has been demonstrated that therapeutics with hydrodynamic size below 12 nm have the most rapid tumor penetration rate.^49^ Smaller AuNPs also demonstrate a longer circulation time *in vivo*, which is beneficial for AuNP accumulation in the tumor.^47^

The radiosensitizing efficacy of AuNPs is associated with many factors, such as particle size, surface coating, and distribution within the cell and tumor.^10^ Previous studies have compared thoroughly the size effects of untargeted AuNPs, ranging from 3 nm to 50 nm on X-ray therapy sensitization and have shown that AuNPs with diameter 13 nm possessed the best enhancement.^17^ Here, we compared PSMA-targeted AuNPs with core sizes of 2 nm, 5 nm and 19 nm, and found that 2 nm PSMA-targeted AuNPs displayed the best radiation enhancement both *in vitro* and *in vivo*, although the 19 nm PSMA-targeted AuNPs showed the highest cellular uptake *in vitro*. There are several reasons why the 2 nm particles, which represent less total cellular gold uptake, may still be the best X-ray sensitizers. First, the size of AuNP, at any given radiation dose, will impact the escape probability of Auger electrons that can be emitted by X-ray irradiated gold. The mean escape depth for gold is 1–5 nm depending on the kinetic energy of the impacted electron. Therefore, larger particles will attenuate the kinetic energy of the freed electrons and may not eject an energetic electron at all, *i.e.* rapid loss of therapeutic enhancement for particles of increasing size.^50^,^51^ Second, it appears that the location of the targeted-AuNP may impact the radiosensitizing efficacy. As evidenced by silver staining, the 2 nm AuNPs had a more homogeneous distribution and deeper penetration into cells and tumors than the 19 nm AuNPs, which may enable severe mitochondrial dysfunction and cellular damage induced by intracellular reactive oxygen species (ROS) upon radiation.^9^ Recently, Yang *et al.* have shown that AuNPs delivered intracellularly *via* amphiphilic lipids are localized more homogeneously resulting in much higher radiation-induced cell killing.^52^ Furthermore, AuNP size has also been shown to impact the location of the particle inside cells. Oh *et al.* has found that cell penetrating peptide (CPP)-targeted 2.4 nm AuNPs were found in the nucleus, while 5.5 and 8.2 nm CPP-AuNPs were found near the nucleus, but did not enter.^53^ Given the short therapeutic distance of the generated auger electrons resulting from irradiated AuNP, close proximity to critical cellular mechanisms likely will improve cellular toxicity. This may be the reason that 2 nm AuNPs are known to have a much higher dose enhancement ratio than any of the other larger NPs.^17^ For *in vivo* radiotherapy, the enhancement is dependent on the amount of Au present in the tumor. With more AuNPs deposited in the tumor, higher radiation dose and therapy enhancement will be facilitated. Therefore, AuNPs with a smaller size are preferred since they have a higher tumor accumulation.^8^ Our observed tumor growth inhibition after radiation therapy agrees with this hypothesis, with the most significant radiation therapy enhancement resulting from 2 nm PSMA-targeted AuNPs.

An additional consideration in developing AuNP-based radiosensitizers is their elimination and metabolism pathways. PEGylated AuNPs are very stable *in vivo*. As has been demonstrated, after intravenous injection, most AuNP are eliminated from the circulation and trapped by the RES, predominantly in the liver, and removed from the body *via* the small intestines.^39^ The AuNPs used in our study with core size of 2 nm, 5 nm, and 19 nm showed an increased liver retention at 24 h post injection, which is in agreement with a previous study by Hirn *et al.*^54^ Smaller particles are more easily cleared from the body, reducing potential liver toxicity.^39^ In our system we have observed that the smaller AuNP, *i.e.* 2 nm AuNPs, had the lowest level of liver uptake and also showed a tendency to be cleared from the urinary system, indicating a possible renal clearance pathway for AuNPs when their size is smaller than the kidney filtration threshold. These observations suggest that smaller AuNPs may have less off-target toxicity if they can be rapidly cleared *via* the urinary system. Their long-term metabolism is still to be investigated.

## Conclusions

PSMA-targeted AuNPs are promising radiosensitizers for radiotherapy of prostate cancer. The PSMA-1 ligand is key for improving specific uptake of AuNPs by PSMA-expressing PC3pip cells regardless of particle size. However, particle size played a key role in the ability of the targeted AuNPs to sensitize cells to radiation therapy. Our studies showed that *in vitro* larger targeted AuNP resulted in more gold uptake into cells, but that the smaller particles were more effective for augmenting radiotherapy of the cells. This might be in part due to the ability of the smaller particles to locate in the vicinity of critical structures within the cells and more effectively sensitize these structures to X-ray therapy. *In vivo*, however, gold uptake into the cells was lower when animals were administered larger AuNPs resulting in the smaller AuNPs being more effective radiosensitizers *in vivo*. It is likely that the smaller AuNP are more effective because of their ability to extravasate from the vasculature to bind and enter into tumor cells, which seemingly occurs less with the 19 nm AuNPs. It appears that smaller AuNPs resulted in lower levels of liver uptake and may lower off-target elemental toxicity of gold and/or radiation sensitivity to non-targeted tissues. These data suggest that small targeted AuNPs are more effective radiosensitizers and studies to further reduce their size are underway.

## Conflicts of interest

There are no conflicts to declare.

## Supplementary Material

Supplementary information
