# Supplementary material for: Prostate-specific membrane antigen targeted gold nanoparticles for prostate cancer radiotherapy: does size matter for targeted particles?
Source: Chem Sci. 2019 Jul 18;10(35):8119–28. doi: 10.1039/c9sc02290b (PMC6764472; doi:10.1039/c9sc02290b)
Supplement: Supplementary file 1 [file SC-010-C9SC02290B-s001.pdf]

Electronic Supplementary Material (ESI) for Chemical Science.
This journal is © The Royal Society of Chemistry 2019

Electronic Supplementary Information

Prostate-specific Membrane Antigen Targeted Gold Nanoparticles for Prostate Cancer Radiotherapy: Does Size Matter for Targeted Particles?

Dong Luo[a], *Xinning Wang*[a,b], *Sophia Zeng*[a,c], *Gopalakrishnan Ramamurthy*[a], *Clemens Burda*[c,*], *James P. Basilion*[a,b,*]

[a]Department of Radiology, Case Western Reserve University, Cleveland, OH

[b]Department of Biomedical Engineering, Case Western Reserve University, Cleveland, OH

[c]Department of Chemistry, Case Western Reserve University, Cleveland, OH

Synthesis of PSMA-1 ligand

Generally, the PSMA-1 synthesis was accomplished by reacting 3.3 equivalents of Fmoc-Lys(Boc)-OH with 0.2 mmol C-terminated Fmoc-rink amide MBHA resin in DMF, with 3.3 and 5 equiv of HCTU and diisopropylethylamine (DIPEA) respectively added as coupling agents. Fmoc was removed using 20% piperidine in DMF before adding the next amino acid. After the sequence of Glu'-Amc-Ahx-Glu-Glu-Glu-Lys(Boc) was conjugated to the resin, the Fmoc of Glu' was removed with 20% piperidine The resin was swollen in chloroform with 2.5 equiv of DIPEA. Then the Glu-COOH pre-conjugation was prepared by mixing 3 equiv of H-Glu(OtBu)-OtBu with 2.5 equiv of DIPEA in chloroform and then adding slowly to 0.25 equiv of triphosgene in chloroform and reacting for 15 min. The reacting mixture was then added to the resin to react for another 45 min. After the reaction, the resin was washed with DMF and methanol respectively, and dried. The peptide was cleaved from the resin with a cleavage cocktail of TFA/H_2O/triisopropylsilane (95%/2.5%/2.5%) and precipitated with cold diethyl ether, and then purified by HPLC. The synthesized PSMA-1 peptides were characterized with electrospray ionization mass spectrometry (ESI-MS, LCQ Advantage, Thermo Finnigan).

Synthesis of SH-PEG$_{2K}$-PSMA-1 conjugation

PSMA-1 and PEG$_{2K}$ (polyethylene glycol) were conjugated via a NH_2 and NHS reaction. PSMA-1 was dissolved in DMF and mixed with 3-fold excess of NHS-PEG$_{2K}$-OPSS in DMF. The pH of the reaction mixture was then adjusted to around 7 with Et_3N, and left on a vortex overnight. The reacted OPSS-PEG$_{2K}$-PSMA-1 conjugation was then purified by HPLC, and then deprotected by adding a 10-fold of tris(2-carboxyethyl)phosphine to remove the OPSS group, yielding the final product SH-PEG$_{2K}$-PSMA-1. The excess tris(2-

carboxyethyl)phosphine was removed by dialysis (MWCO=2kDa) against H_2O. The final product was lyophilized and stored in -20 °C for future use. High-resolution matrix-assisted laser desorption/ionization−time-of-flight mass spectroscopy (MALDI-TOF MS, Bruker AutoFlex III) was used for characterization.

Synthesis and characterization of gold nanoparticles

Gold nanoparticles with three different sizes were synthesized. AuNPs with core size of 2 nm and 5 nm were synthesized in a two-phase toluene-H_2O system. For 2 nm AuNPs, 80 mg of $HAuCl_4$•$3H_2O$ and 260 mg of tetraoctylammonium bromide (TOAB) were dissolved in 5 ml H_2O and 14 ml toluene respectively, and the two phases were mixed and stirred for 5 min. Then 18 mg DDA was added to the mixture and stirred for another 10 min. 5 ml of 13 mg/ml sodium borohydride ($NaBH_4$) ice-cold H_2O solution was then added in very quickly (within 10s). The reaction mixture was stirred for more than 4 hours. For 5 nm AuNPs synthesis, 136.7 mg TOAB was dissolved in 5 ml toluene and 367 ul of gold(III) chloride solution (30 wt % in dilute HCl) was added and stirred for 5 min. 112 mg DDA was then added to the mixture. Following another 10 min of stirring, 1 ml of 75.6 mg/ml $NaBH_4$ ice-cold H_2O solution was added dropwise. The reaction was then carried on for 2 hours. The produced AuNPs were then precipitated in pure ethanol and centrifuged at 4500 rpm for 10 min. The supernatants were decanted and AuNPs were washed again with pure ethanol. The precipitations were dried under N_2 gas stream and then dissolved in chloroform and centrifuged again at 4500 rpm for 10 min. The supernatants were collected and measured by UV-vis spectroscopy to determine the concentration.

AuNPs with core size of 19 nm were synthesized using the Turkevich method [1]. Generally, 100 ml of 0.01% $HAuCl_4$•$3H_2O$ solution was refluxed and added with 2.5 ml of

1% sodium citrate solution was added under vigorous stirring. A color change from yellow to red would occur in 5 min. The heat source was removed after 30 min reacting and the solution was cooled down to room temperature.

The concentration of the AuNP samples was determined by UV−vis spectroscopy based on the plasmonic absorption band at 520 nm. To functionalize AuNPs with SH-PEG_{2K}-PSMA-1, a 1000 molar excess of SH-PEG_{2K}-PSMA-1 and SH-PEG_{2K} ligands at different molar ratio (1:8, 1:4, 1:1 to 2:1) were added to react with 1 equiv of AuNP-DDA or AuNP-citric acid for 2 days. Excess unreacted ligands were removed by extensive purification using centrifuge filters (MWCO = 30 kDa, GE Healthcare). As control, non-targeted AuNPs were also prepared in the same way with only SH-PEG_{2K} added and purified.

The hydrodynamic size of AuNPs was characterized with a dynamic light scattering system (DynoPro NanoStar). The zeta potential of AuNPs was measured with a Zetasizer Nano (Malvern). For absolute size determination, transmission electron microscopy (FEI Tecnai F300 kV). Samples were prepared by putting one drop of sample solution onto 400 mesh formvar/carbon supported copper grids (Ted Pella, Inc.), and dried naturally at room temperature. To visualize the polymer shells, the nanoparticles loaded copper grids were stained by one drop of 2% Phosphotungstic acid for 5 min and then the excess liquid was wicked off with filter paper. The grids after staining were dried again in air before TEM testing. Besides, gel electrophoresis for all the PSMA-targeted and untargeted nanoparticles with various sizes were performed on 1% agarose gel and 1× TAE running buffer at 120 kV. Each chamber was loaded with 10 μL of 2 uM AuNPs, 5 μL of glycerol, and 5 μL of 4× TAE.

Reference

[1] C. Spaas , R. Dok, O, Deschaume, B. De Roo, M. Vervaele, J. W. Seo, Dependence of gold nanoparticle radiosensitization on functionalizing layer thickness, *Radiat Res.* 185 (2016) 384-92.

a

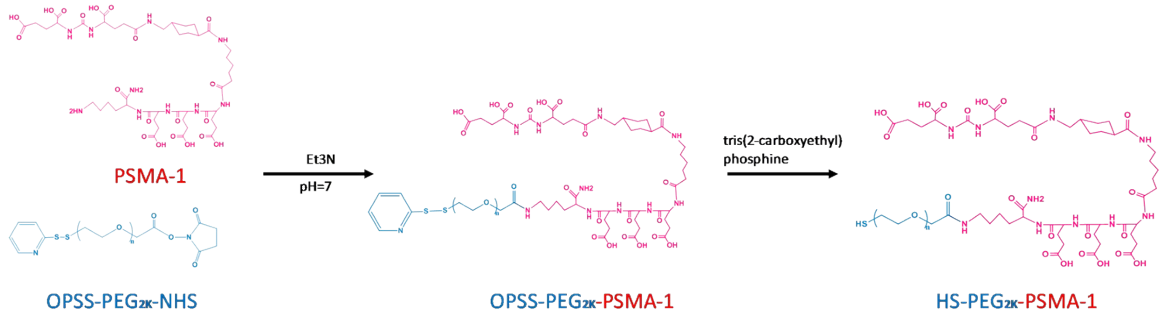

b

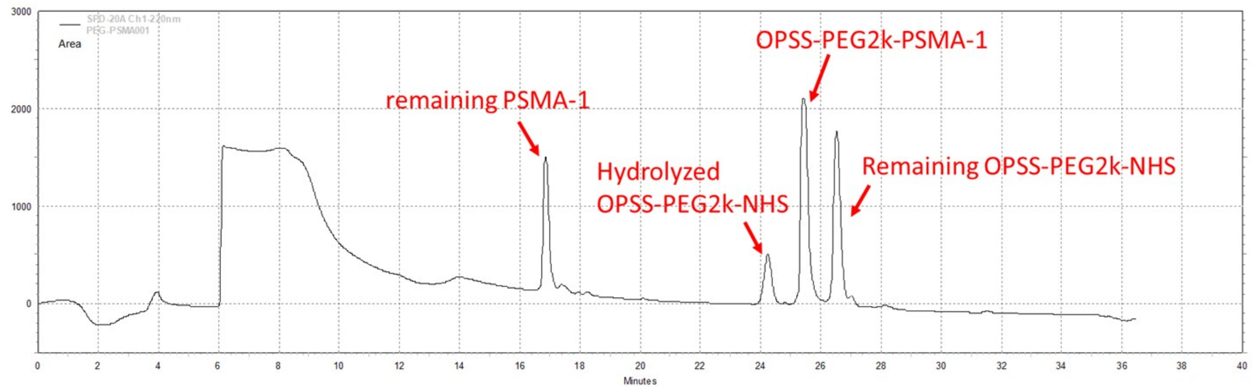

Fig. S1 (a) Synthesis of PEG2k-PSMA-1 in two steps: first, OPSS-PEG$_{2k}$-NHS was reacted with PSMA-1 and then –SH was deprotected producing HS-PEG$_{2k}$-PSMA-1. (b) HPLC purification of reaction mixture from the first step.

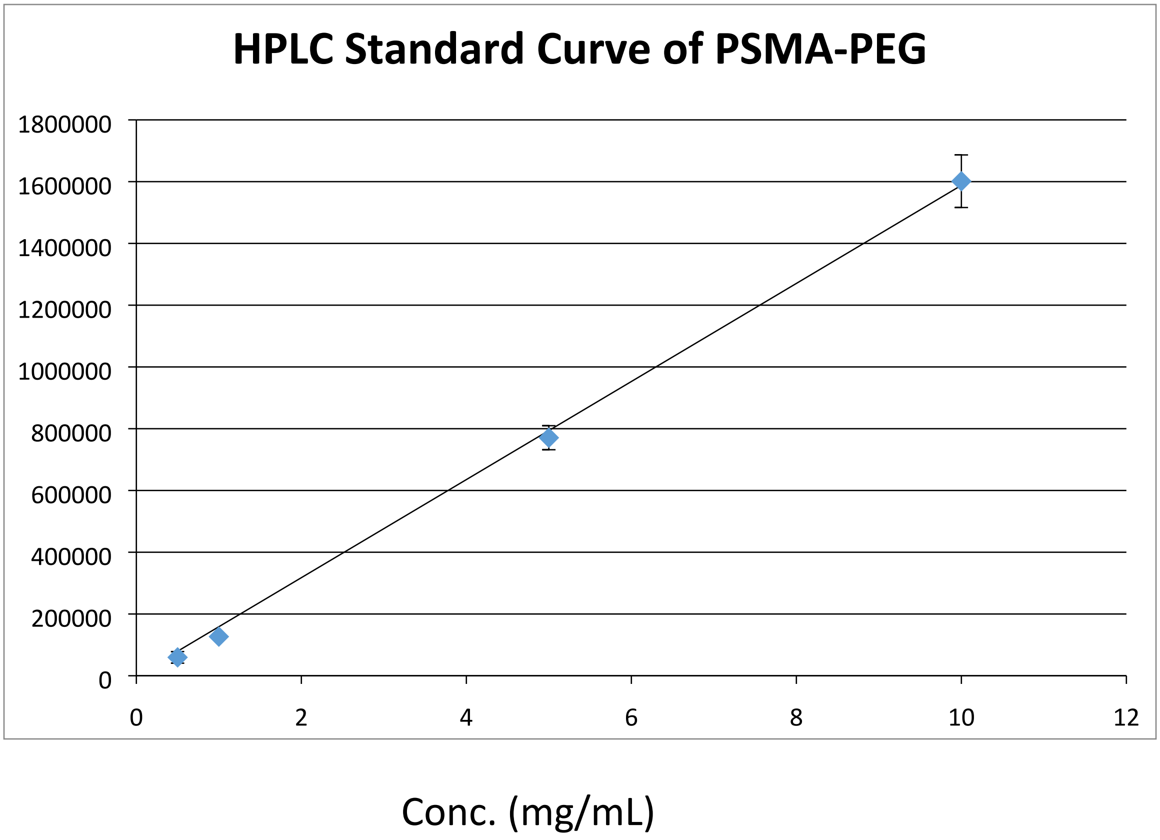

Fig. S2 HPLC standard curve of PSMA-PEG which is used for determining remaining PEG-PSMA-1 from AuNPs conjugation.

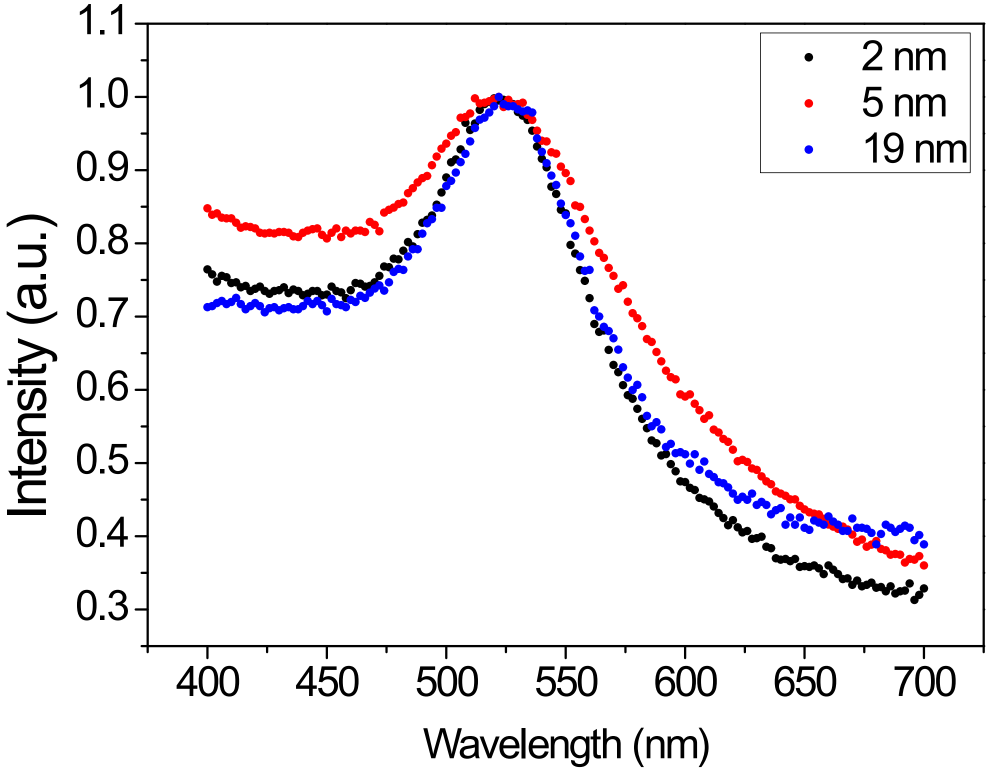

Fig. S3 UV-vis absorption spectra of 2 nm, 5 nm and 19 nm AuNPs.

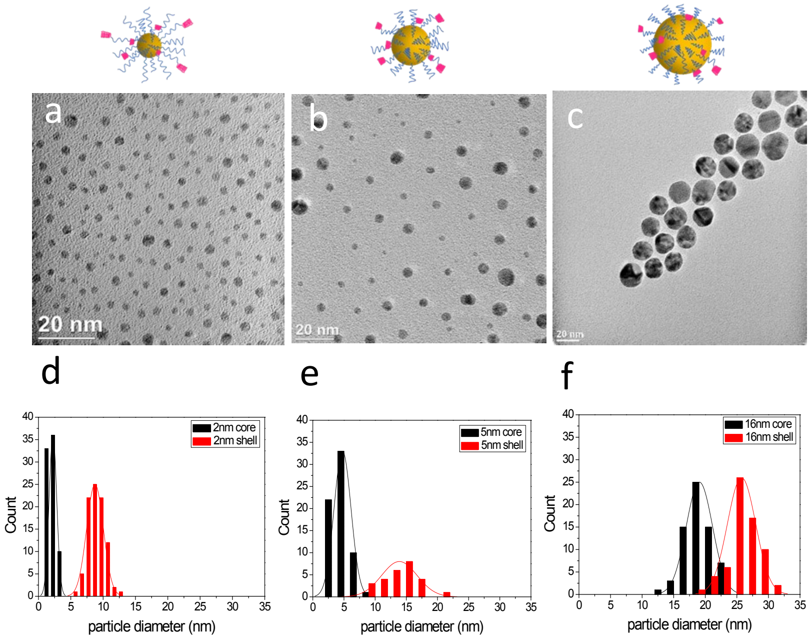

Fig. S4 Characterization of AuNP sizes. (a-c) TEM images AuNPs with average core sizes of (a) 2nm, (b) 5 nm and (c) 19 nm, and (d-f) Size distributions of AuNPs core size from TEM (black) and hydrodynamic size measured from DLS (red).

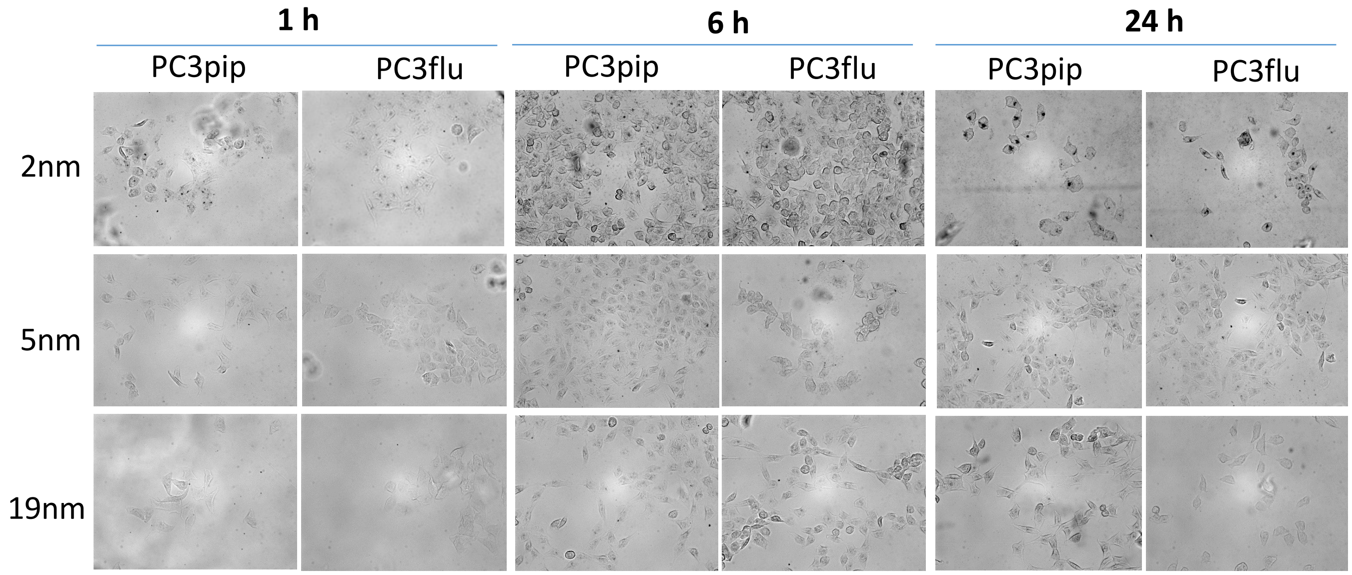

Fig. S5 Cell uptake of untargeted AuNPs with different sizes. Untargeted AuNPs with core sizes of 2 nm, 5 nm and 19 nm were incubated with PC3pip and PC3flu cells for 1 h, 6 h and 24 h and then stained with silver staining kits. Silver nucleates around the AuNPs and thus reveals the AuNP uptake by the cells.

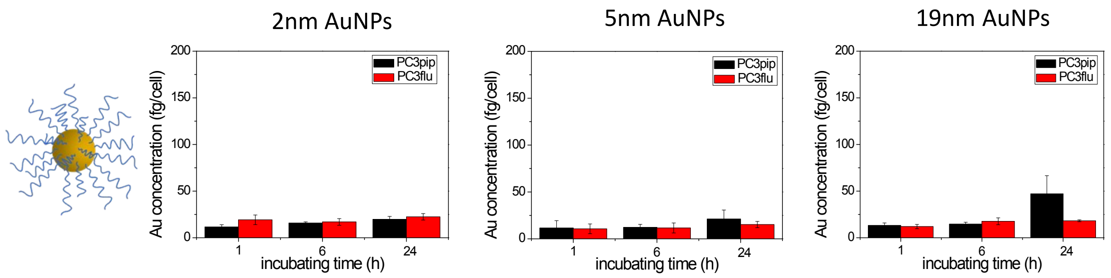

Fig. S6 Effect of particle size on cell uptake of untargeted AuNPs measured with ICP-MS. PC3pip and PC3flu cells incubated with untargeted AuNPs, were trypsinized, washed, and digested. Au content in each cell was determined by ICP-MS and divided by total cell numbers. Data are presented as mean±SD (n = 3).

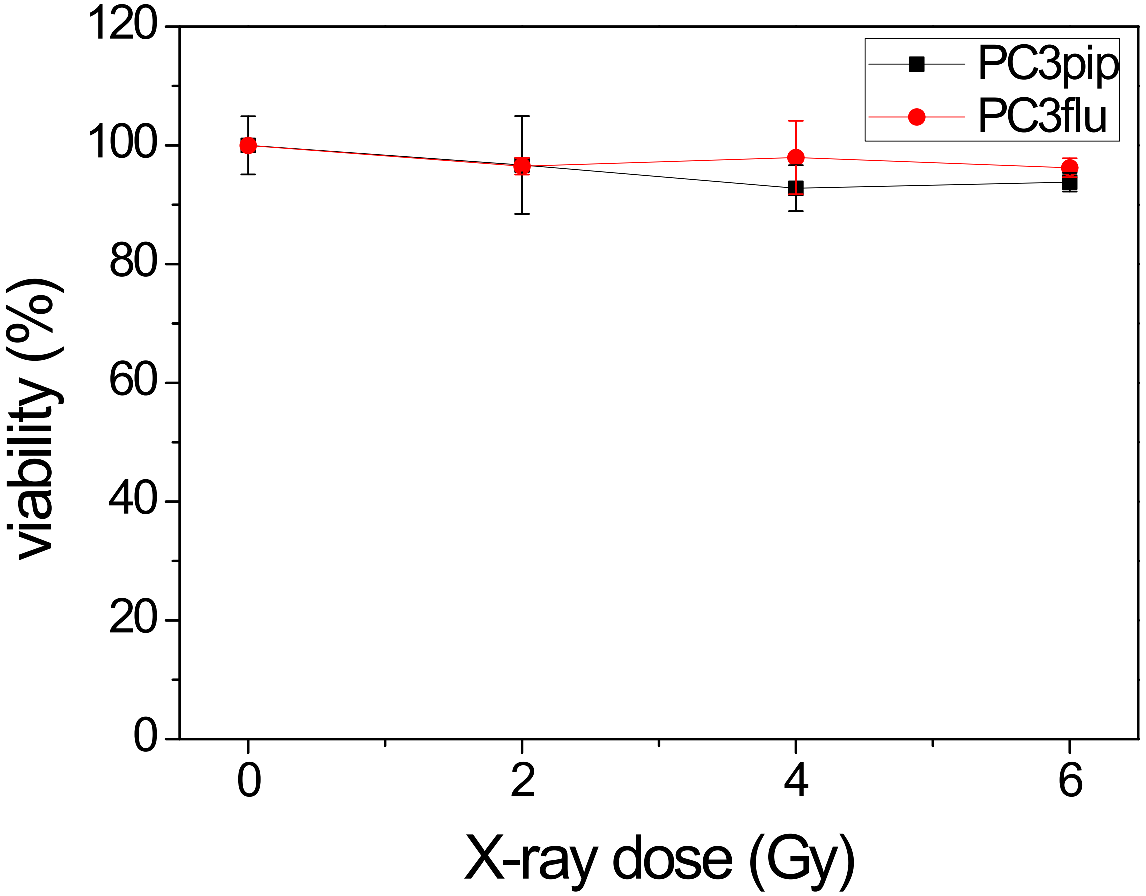

Fig. S7 Cell viability of PC3pip and PC3flu cells without AuNPs added and irradiated with different doses of X-ray radiation. Data are presented as mean±SD (n = 3). Irradiation without AuNPs is ineffective in cell killing.

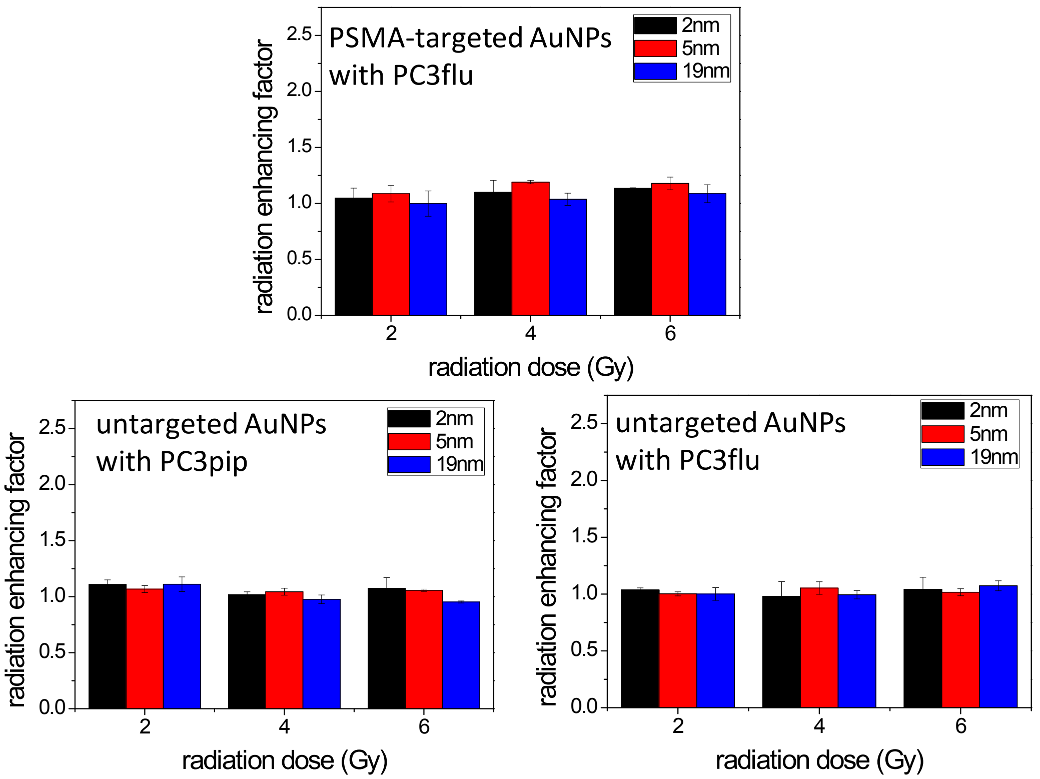

Fig. S8 Radiation enhancing factor (REF, ratio of eradicated cells with and without AuNPs) for PC3pip and PC3flu cells incubated with PSMA-targeted AuNPs and untargeted AuNPs at radiation doses of 2 Gy, 4 Gy and 6 Gy. The gold concentrations were kept constant for each sample. Data are presented as mean± SD (n = 3).

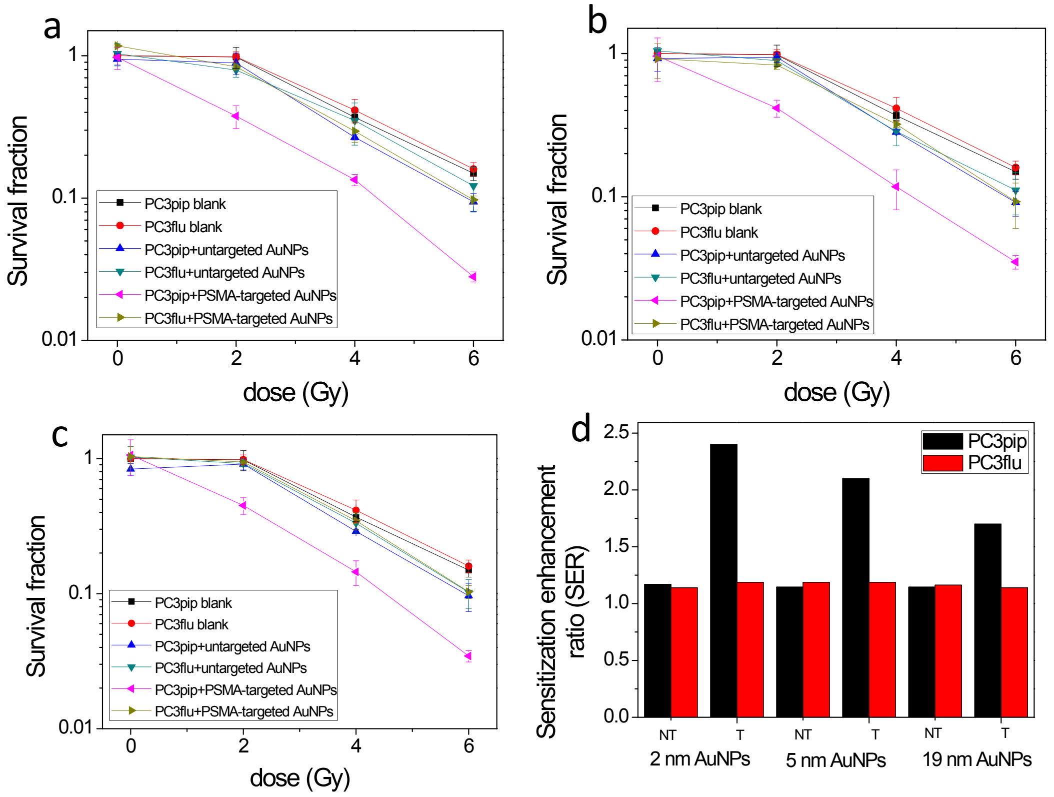

Fig. S9 Colony formation assay for PC3pip and PC3flu cells incubated with PSMA-targeted and untargeted AuNPs with sizes of (a) 2 nm, (b) 5 nm, and (c) 19 nm at radiation doses of 2 Gy, 4 Gy and 6 Gy, and (d) the according sensitization enhancement ratio (SER) for each of the particles. The gold concentrations were kept constant for each sample. Data are presented as mean± SD (n = 3).

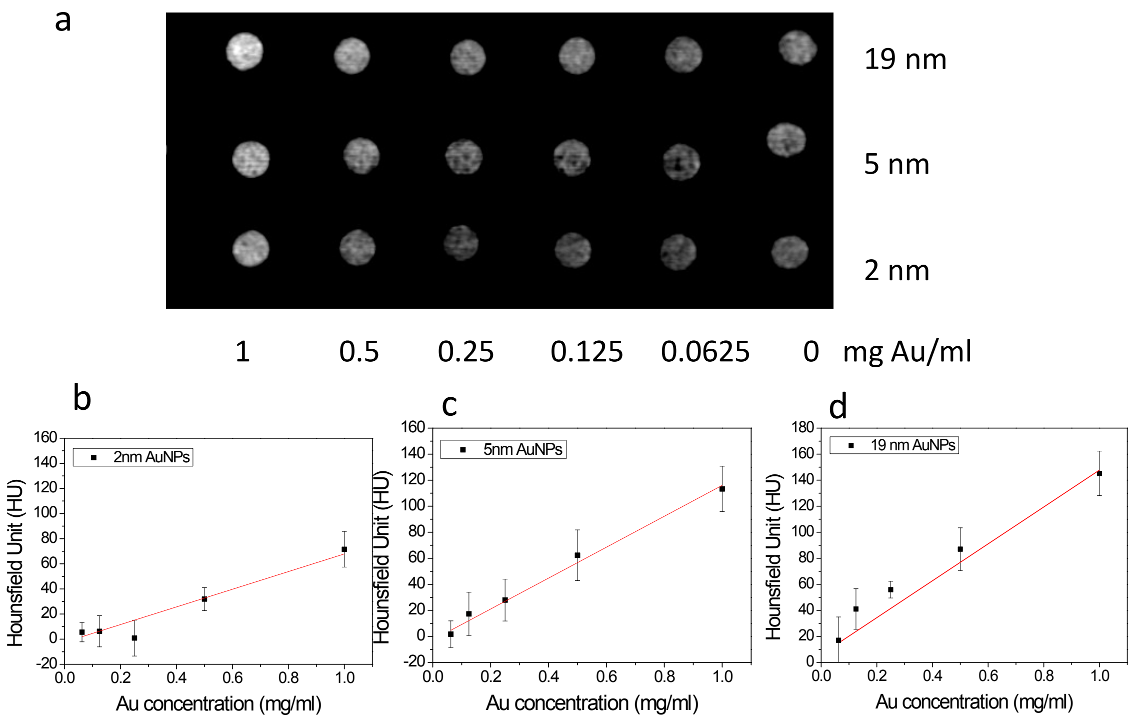

Fig. S10 (a) CT images of phantoms containing AuNPs with size of 2 nm, 5nm and 19 nm at various concentrations. (b-d) X-ray attenuation intensity for 2 nm, 5 nm and 19 nm AuNPs as a function of concentration of gold. The data shows good linearity as function of Au concentration. Data are presented as mean± SD (n = 3).

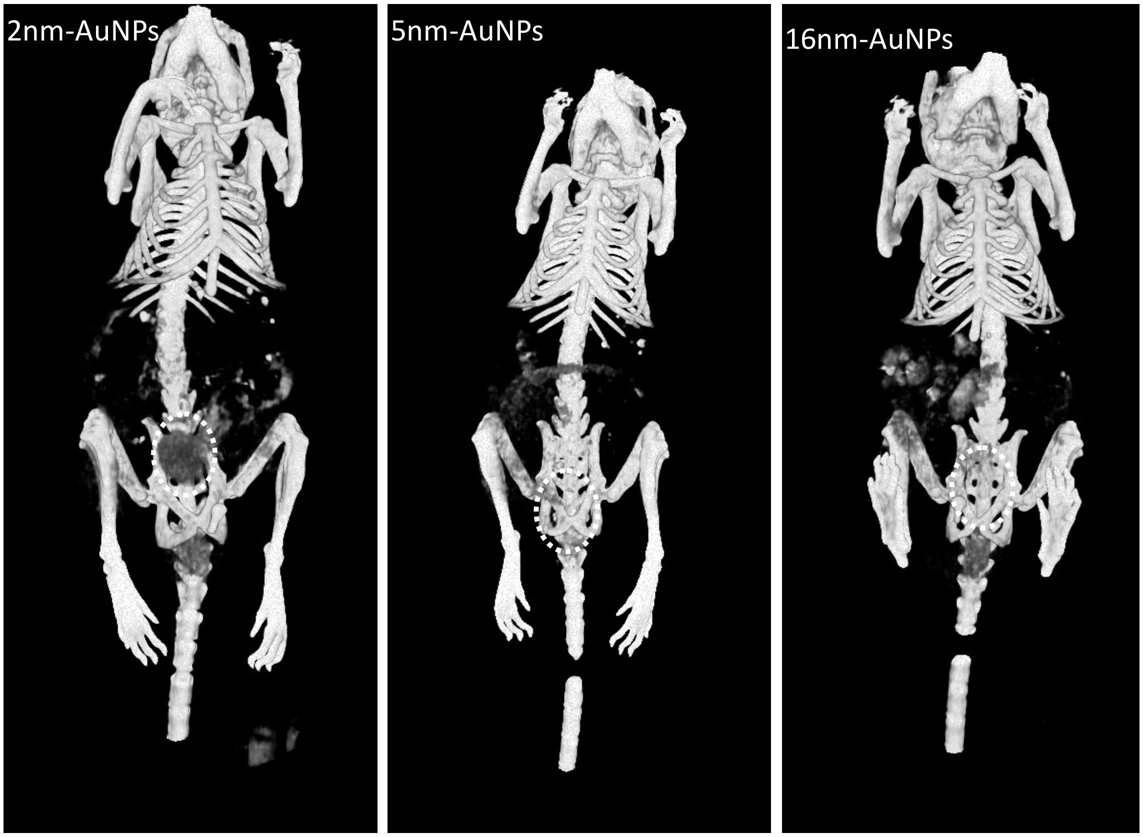

Fig. S11 *In vivo* 3D CT images of bladders (indicated by dash circles) after injection of 2 nm, 5 nm and 19 nm AuNPs. Mouse injected with 2 nm AuNPs showed enhanced CT signal in bladder, indicating urinary excretion of AuNPs, while mice injected with 5 nm and 19 nm AuNPs did not show signal enhancement in bladders.

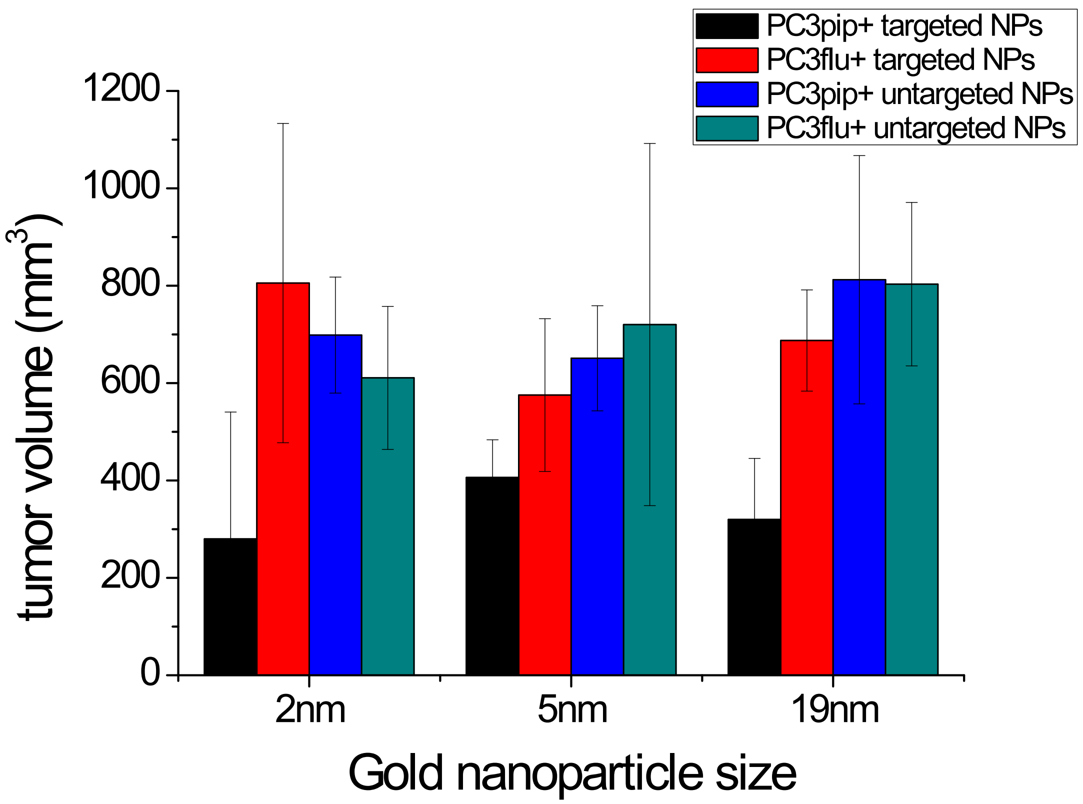

Fig. S12 PC3pip and PC3flu tumor volume at day 20 after injection of PSMA-targeted and untargeted AuNPs with different sizes and irradiated at 6 Gy. Data are presented as mean±SD (n = 3).

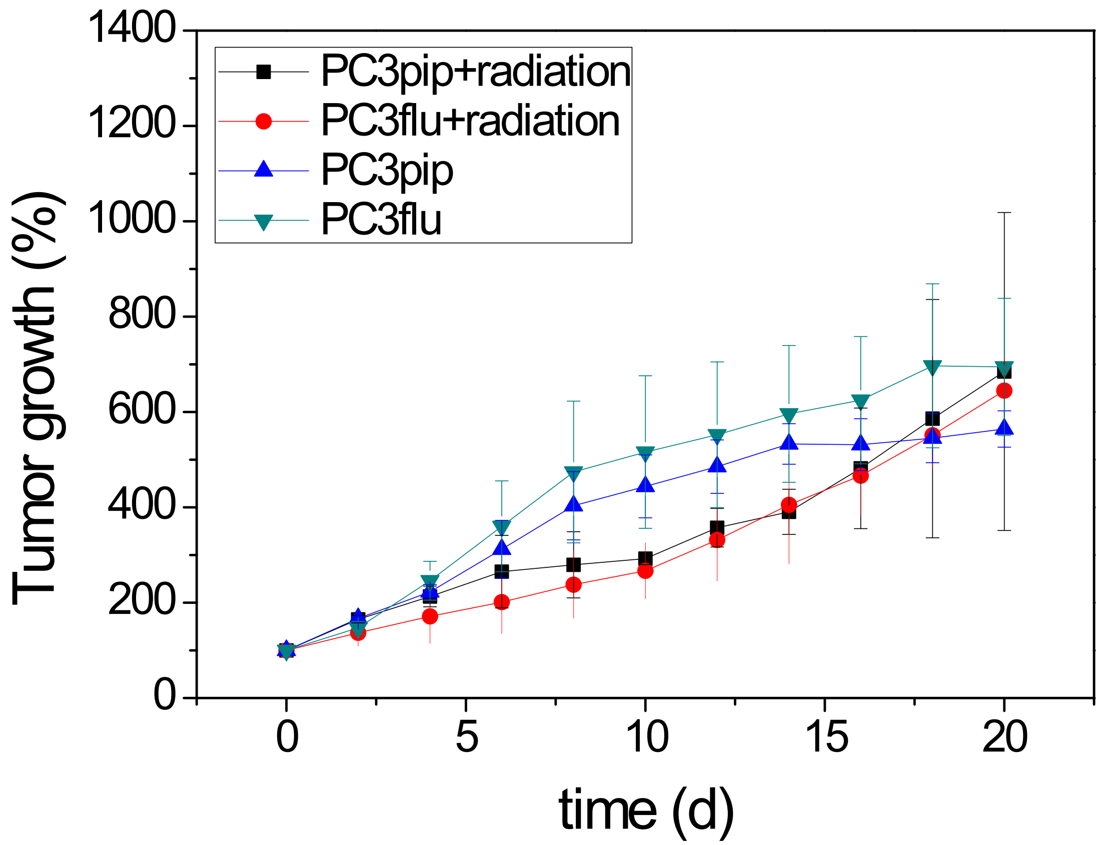

Fig. S13 PC3pip and PC3flu tumor growth curves after injection of PBS and X-ray radiation at 6 Gy. Data are presented as mean± SD (n = 3).

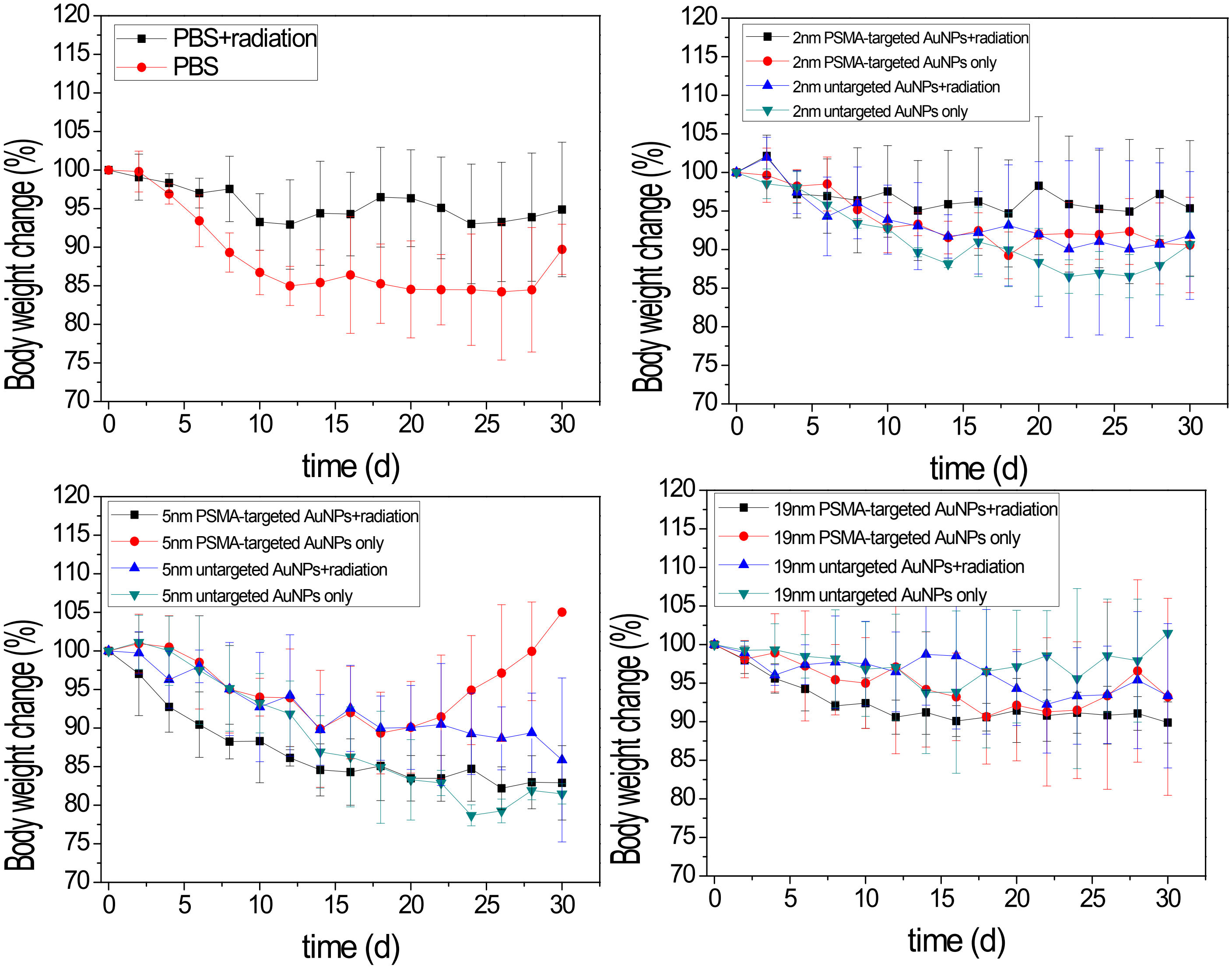

Fig. S14 Body weight changes of mice after injection of PBS, PSMA-targeted and untargeted AuNPs of different sizes.
